# A Hemodynamic Bridge from Echocardiography to Directly Measured Left Ventricular End-Diastolic Pressure: The Intermediate Role of Pulmonary Artery Diastolic Pressure in a Routine Catheterization Cohort

**DOI:** 10.3390/diagnostics16101559

**Published:** 2026-05-20

**Authors:** Aykan Çelik, Tuncay Kiris, Harun Erdem, Semih Babacan, Cem Nazlı

**Affiliations:** 1 Department of Cardiology, Izmir Atatürk Training and Research Hospital, Izmir 35360, Türkiye; 2 Department of Cardiology, Medicine Faculty, Izmir Katip Çelebi University, Izmir 35620, Türkiye

**Keywords:** eft ventricular end-diastolic pressure, pulmonary artery diastolic pressure, echocardiography, pulmonary hemodynamics, cardiac catheterization, pulmonary capillary wedge pressure, filling pressure, diagnostic modeling

## Abstract

**Background**: Noninvasive echocardiographic markers are widely used to estimate left ventricular filling pressure, but their relationship with directly measured left ventricular end-diastolic pressure (LVEDP) is often modest and context-dependent. Whether routinely available noninvasive findings reflect elevated LVEDP through an intermediate invasive pulmonary hemodynamic phenotype remains insufficiently characterized. **Objective**: To evaluate the relationship of noninvasive echocardiographic and laboratory markers with directly measured LVEDP and to determine whether invasive pulmonary artery diastolic pressure (dPAP) functions as a hemodynamic bridge linking upstream noninvasive findings to elevated left ventricular filling pressure in a routine catheterization cohort. **Methods**: This retrospective single-center observational study included patients undergoing routine cardiac catheterization with available direct LVEDP measurement and invasive pulmonary artery pressure data. Elevated LVEDP was defined as LVEDP ≥ 15 mmHg, and elevated dPAP as dPAP ≥ 24 mmHg. Noninvasive, bridge, and invasive validation models were evaluated using logistic regression, receiver operating characteristic analysis, calibration assessment, and bootstrap internal validation. **Results:** A total of 75 patients had direct LVEDP data, 94 had invasive dPAP data, 83 had echocardiographic systolic pulmonary artery pressure (echo-sPAP), and 37 had pulmonary capillary wedge pressure (PCWP) measurements. Patients with elevated LVEDP had significantly higher creatinine (*p* = 0.026), dPAP (*p* = 0.043), and PCWP (*p* = 0.004). Echo-sPAP showed good discrimination for elevated dPAP, with an AUC of 0.791 (95% CI 0.695–0.888), supporting its role as an upstream noninvasive marker of invasive pulmonary hemodynamic burden. A noninvasive model combining echo-sPAP and creatinine showed modest discrimination for elevated LVEDP (AUC 0.664, 95% CI 0.522–0.806; Brier score 0.198), whereas an invasive validation model combining dPAP and creatinine showed better performance (AUC 0.734, 95% CI 0.617–0.850; Brier score 0.176). In bootstrap validation, the optimism-corrected AUCs were approximately 0.624 and 0.711, respectively. Although the invasive model performed numerically better, DeLong comparison did not show a statistically significant difference between the two models (*p* = 0.459). **Conclusions**: Routinely available noninvasive echocardiographic and laboratory findings appear to relate to directly measured left ventricular filling pressure through an intermediate invasive pulmonary hemodynamic pattern. Echo-sPAP showed its strongest signal at the level of elevated dPAP, whereas dPAP combined with creatinine provided the most informative model for elevated directly measured LVEDP. These findings support a hypothesis-generating hemodynamic framework linking noninvasive assessment to directly measured filling pressure and may help inform noninvasive hemodynamic triage and physiological risk enrichment in selected clinical settings.

## 1. Introduction

Accurate assessment of left ventricular filling pressure remains a central challenge in cardiovascular medicine. Elevated left ventricular end-diastolic pressure (LVEDP) is clinically significant across a broad spectrum of cardiac disorders, yet direct invasive measurement is rarely feasible outside catheterization laboratories. Consequently, noninvasive echocardiographic markers are widely used to estimate filling pressures in clinical practice [1]. However, the association between echocardiographic parameters and invasively measured filling pressure is often imperfect, varies across patient populations, and may be influenced by underlying hemodynamic complexity.

Current echocardiographic methods primarily rely on Doppler-based indices and surrogates of pulmonary pressure to estimate left-sided filling pressures. Although these tools provide valuable clinical insights, their performance is typically moderate rather than definitive, and discordance between noninvasive estimates and invasive measurements remains common [2,3]. Furthermore, many prior studies have used pulmonary capillary wedge pressure (PCWP) as a reference instead of directly measured LVEDP, a distinction of physiological importance because PCWP and LVEDP, although related, are not interchangeable in all conditions [4,5,6,7]. A conceptual comparison of established noninvasive and invasive approaches, together with the proposed dPAP bridge framework, is provided in Supplementary Table S12. In routine catheterization practice, elevated left-sided filling pressure may be transmitted to the pulmonary circulation and become partially reflected in right-sided or pulmonary artery hemodynamics [8,9]. In this context, pulmonary artery diastolic pressure (dPAP) may serve as an intermediate parameter linking noninvasive findings with directly measured LVEDP [8]. Yet this multilayered relationship—integrating noninvasive echocardiography, laboratory markers, invasive pulmonary hemodynamics, and direct LVEDP data within the same cohort—has not been systematically examined.

Therefore, rather than reassessing a direct correlation between echocardiographic indices and LVEDP, we aimed to evaluate whether routinely available noninvasive and laboratory markers relate to LVEDP through an intermediate invasive pulmonary hemodynamic pathway. Specifically, we evaluated whether echocardiographic estimates of pulmonary pressure are associated with invasive dPAP and whether dPAP, in turn, correlates with elevated LVEDP.

## 2. Methods

### 2.1. Study Design and Population

This retrospective, single-center, observational study included consecutive patients who underwent routine cardiac catheterization and had direct left ventricular end-diastolic pressure (LVEDP) measurement available during the procedure. Patients with available invasive pulmonary artery pressure recordings were considered for the main analytical cohort. The study was designed to evaluate the relationship of routinely available noninvasive echocardiographic and laboratory markers with directly measured LVEDP and to investigate whether invasive pulmonary artery diastolic pressure (dPAP) functions as an intermediate hemodynamic bridge linking upstream noninvasive findings to elevated left ventricular filling pressure.

Patients were eligible for inclusion if direct LVEDP measurement and at least one invasive pulmonary artery pressure parameter were available from the same catheterization episode. Patients with missing LVEDP data, absent pulmonary artery pressure data, technically unreliable hemodynamic recordings, or repeated procedures without a clearly defined index examination were excluded from the corresponding analyses. When more than one catheterization record was present, the first eligible examination was used as the index procedure. Because data availability differed across clinical, echocardiographic, and invasive variables, the number of observations varied between descriptive analyses and model-specific complete-case datasets. Missingness primarily reflected the retrospective availability of routinely collected variables, particularly for echocardiographic indices and PCWP. Given the modest sample size, variable-specific availability patterns, and the exploratory physiologically oriented design of the study, complete-case analysis was retained as the primary analytical approach.

### 2.2. Data Collection

Clinical, laboratory, echocardiographic, and invasive hemodynamic data were retrospectively extracted from the institutional database and catheterization records. Demographic data, routine laboratory parameters, transthoracic echocardiographic measurements, and invasive pressure recordings obtained during routine clinical care were collected. Variables considered for the analysis were selected on the basis of physiological plausibility, routine clinical availability, and data completeness.

The primary noninvasive variables of interest were echocardiographic systolic pulmonary artery pressure (echo-sPAP), tricuspid regurgitation maximum velocity (TR Vmax), serum creatinine, and hemoglobin. Estimated glomerular filtration rate (eGFR) was calculated from serum creatinine, age, and sex using the 2021 Chronic Kidney Disease Epidemiology Collaboration (CKD-EPI) creatinine equation, which does not include race as a coefficient. eGFR was used in a sensitivity analysis, replacing serum creatinine, to assess whether the observed associations were dependent on the renal function metric used [10]. Additional exploratory noninvasive variables included left ventricular ejection fraction (LVEF), left atrial diameter, blood urea nitrogen, white blood cell count, and available diastolic echocardiographic indices. The principal invasive variables of interest were pulmonary artery systolic pressure (sPAP), pulmonary artery diastolic pressure (dPAP), mean pulmonary artery pressure (mPAP), pulmonary capillary wedge pressure (PCWP) when available, and directly measured LVEDP.

### 2.3. Echocardiographic Assessment

Echocardiographic data were derived from routine transthoracic echocardiographic examinations performed as part of standard clinical evaluation. Measurements were based on the original echocardiographic reports and archived clinical records. Echo-sPAP and TR Vmax were considered the main echocardiographic variables in the present study because they were the most consistently available noninvasive markers related to pulmonary hemodynamics. Echo-sPAP was abstracted from the original transthoracic echocardiography reports. In routine echocardiographic reporting, sPAP is estimated from the peak tricuspid regurgitation velocity using the modified Bernoulli equation, 4 × (TR Vmax)^2^, with the addition of estimated right atrial pressure. Right atrial pressure was estimated by the interpreting echocardiographer according to routine echocardiographic assessment, primarily using inferior vena cava size and collapsibility when available. Because this was a retrospective report-based study, the individual components used to derive right atrial pressure were not consistently available for independent recalculation; therefore, the reported echo-sPAP value was used for analysis.

Other echocardiographic variables, including LVEF and selected chamber dimensions or diastolic indices, were evaluated as supportive or exploratory variables depending on data completeness. Given the retrospective design, echocardiographic examinations performed within 1 month of catheterization were included to balance temporal proximity with data availability. Because hemodynamic status may change within this interval, an additional sensitivity analysis was conducted among patients with echocardiography and catheterization performed within 7 days.

The study was not designed as a revalidation of a single diastolic echocardiographic algorithm. Rather, echocardiographic parameters were examined as clinically accessible upstream markers in relation to invasive pulmonary hemodynamics and directly measured LVEDP.

### 2.4. Invasive Hemodynamic Assessment

Invasive hemodynamic data were obtained during routine cardiac catheterization. Direct LVEDP measured during catheterization was used as the reference marker of left ventricular filling pressure. Invasive pulmonary artery systolic, diastolic, and mean pressures were extracted from the procedural records. Among the invasive pulmonary artery pressure metrics, dPAP was selected as the principal invasive bridging variable based on physiological plausibility and its consistent signal in the exploratory descriptive analyses.

In the present study, mPAP was treated as a derived hemodynamic variable and expressed using the standard approximation, mPAP = (sPAP + 2 × dPAP)/3, based on the recorded systolic and diastolic pulmonary artery pressures [11]. The original sPAP and dPAP values were used as documented. Because the principal invasive bridging analyses were based on dPAP, mPAP served only as a supportive variable.

### 2.5. Outcome Definitions

The primary outcome was elevated LVEDP, defined as direct LVEDP ≥ 15 mmHg. Direct LVEDP was also evaluated as a continuous variable in exploratory analyses.

The secondary invasive intermediate outcome was elevated dPAP, defined as dPAP ≥ 24 mmHg. This threshold was used to operationalize an intermediate invasive pulmonary hemodynamic phenotype for the bridge-model analyses. It was selected as a pragmatic analytical cut point near the cohort median, allowing balanced subgrouping, rather than as a formal disease-defining threshold. We deliberately did not use the ROC-optimized threshold for LVEDP discrimination in order to avoid circular definition of the intermediate phenotype. PCWP was not substituted for LVEDP in any analysis. Patients without direct LVEDP measurement were not included in the primary LVEDP outcome analyses. PCWP, when available, was evaluated only as a supportive subgroup variable to provide contextual hemodynamic information and to illustrate its relationship with directly measured LVEDP.

### 2.6. Prespecified Analytical Sequence and Model Definitions

The analyses followed a prespecified sequential structure rather than a previously established named modeling method. The terms “bridge model,” “noninvasive model,” and “invasive validation model” were used as descriptive, study-specific analytical labels to improve readability of the analytical sequence and were not intended to denote previously validated clinical prediction models.

For clarity, the study-specific model labels were defined as follows. The bridge model evaluated whether an upstream noninvasive pulmonary pressure estimate, echo-sPAP, identified the intermediate invasive pulmonary hemodynamic phenotype, elevated dPAP. The noninvasive LVEDP model evaluated whether clinically available pre-catheterization variables, primarily echo-sPAP and creatinine, were associated with elevated directly measured LVEDP. The invasive validation model evaluated whether invasive dPAP, alone or in combination with creatinine, was associated with elevated directly measured LVEDP. Thus, invasive dPAP was analyzed as an intermediate physiological marker linking noninvasive pulmonary pressure estimates to direct LV filling pressure, not as a bedside post-catheterization prediction score.

### 2.7. Statistical Analysis

Continuous variables were expressed as mean ± standard deviation or median (interquartile range), depending on distributional characteristics. Categorical variables were expressed as counts and percentages. Normality was assessed using visual methods and distributional testing as appropriate.

For comparisons according to the primary outcome, patients were stratified as LVEDP < 15 mmHg and LVEDP ≥ 15 mmHg. Continuous variables were compared using the independent-samples *t* test or Mann–Whitney U test, as appropriate. Categorical variables were compared using the chi-square test or Fisher’s exact test.

Correlations between noninvasive, invasive, and direct LVEDP variables were assessed primarily using Spearman correlation coefficients, given the expected non-normal distribution of several hemodynamic parameters. Correlation analyses focused on the relationships among echo-sPAP, TR Vmax, dPAP, PCWP, and directly measured LVEDP.

Binary logistic regression analyses were performed to identify predictors of elevated dPAP and elevated LVEDP. Model development followed a parsimonious strategy because of the sample size and the number of available events.

Separate model groups were prespecified:

(1) noninvasive clinical models for elevated LVEDP, including echo-sPAP alone and in combination with serum creatinine and selected supportive laboratory variables;
(2) bridge models for elevated dPAP, including echo-sPAP alone and in combination with serum creatinine, with TR Vmax-based models evaluated as sensitivity analyses;
(3) invasive validation models for elevated LVEDP, including dPAP alone and in combination with serum creatinine.

As a sensitivity analysis, multivariable models including renal function were repeated after replacing serum creatinine with eGFR calculated using the 2021 CKD-EPI creatinine equation.

To reduce overfitting, only a limited number of clinically plausible and data-supported variables were entered into each multivariable model. Variables with extensive missingness or uncertain measurement consistency were not included in the primary multivariable analyses.

Model discrimination was assessed using receiver operating characteristic (ROC) curve analysis and area under the curve (AUC). Calibration was evaluated using calibration plots and the Brier score where appropriate. Internal validation was planned using bootstrap resampling. Comparisons between selected ROC curves were performed using DeLong testing when statistically appropriate and feasible given the sample size. Because of variable-specific missingness, sample size differed across descriptive, regression, ROC, and calibration analyses.

As a sensitivity analysis addressing temporal alignment between noninvasive and invasive measurements, the principal models were repeated in the subgroup of patients whose echocardiography and catheterization assessments were performed within 7 days.

Complete-case analysis was used for each individual model. The number of observations included in each analysis was reported explicitly. As an additional sensitivity analysis for missing data, multiple imputation by chained equations was performed for the principal model variables with moderate missingness. Outcomes were not imputed; imputed datasets were used to reassess the principal logistic regression models. Variables with substantial or potentially structural nonavailability, including PCWP and TR Vmax, were not imputed. Twenty imputed datasets were generated using predictive mean matching for continuous variables and logistic regression for binary variables. A two-sided *p* value < 0.05 was considered statistically significant. Statistical analyses were performed using SPSS (Statistical Package for the Social Sciences) version 30.0 (IBM Corp., Armonk, NY, USA) and the R programming language (R Foundation for Statistical Computing, Vienna, Austria, version 4.5.2) within the RStudio environment (Posit PBC, Boston, MA, USA, version 2026.04.0).

The manuscript was prepared in accordance with the STROBE reporting guideline [12]. The completed STROBE checklist is provided as Supplementary File.

### 2.8. Ethical Considerations

The study protocol was reviewed and approved by the local institutional ethics committee. Patient consent was waived by the institutional review board because of the retrospective design and use of de-identified data. The study was conducted in accordance with the principles of the Declaration of Helsinki.

## 3. Results

### 3.1. Study Population and Analytical Cohorts

**Figure 1:**
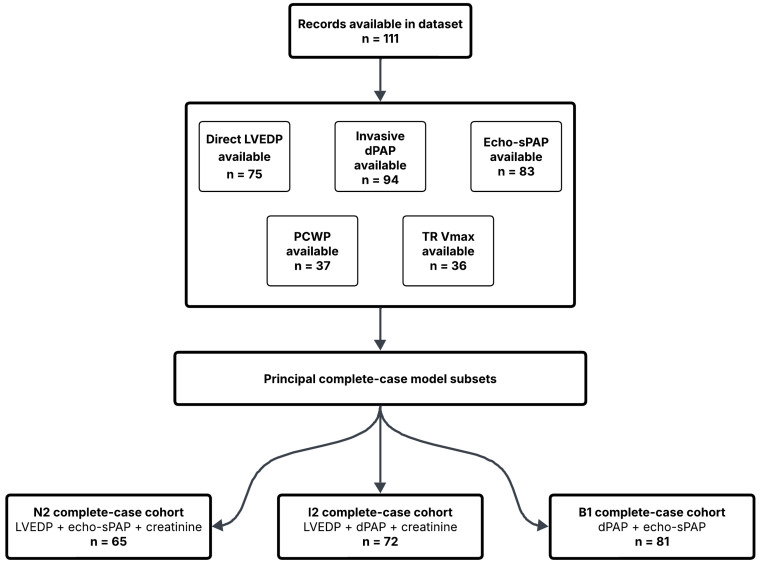
Study flow diagram and analytical cohorts. Flow diagram showing variable availability within the dataset and the complete-case analytical subsets used for the principal models. Of 111 available records, direct LVEDP was available in 75 patients, invasive dPAP in 94, echocardiographic systolic pulmonary artery pressure (echo-sPAP) in 83, TR Vmax in 36, and PCWP in 37. The complete-case subsets used for the principal noninvasive model (N2), invasive validation model (I2), and bridge model (B1) are also shown.

A total of 111 records were available in the working dataset. Direct LVEDP measurement was available in 75 patients, invasive dPAP in 94, invasive sPAP in 95, mPAP in 94, PCWP in 37, echo-sPAP in 83, and TR Vmax in 36 patients. Serum creatinine and hemoglobin were available in 97 patients each. The primary binary outcome, elevated LVEDP (LVEDP ≥ 15 mmHg), was evaluable in 75 patients, whereas the intermediate invasive pulmonary hemodynamic phenotype, elevated dPAP (dPAP ≥ 24 mmHg), was evaluable in 94 patients. Among patients with available direct LVEDP measurement, elevated LVEDP was present in 55 of 75 patients (73.3%). The study flow and the complete-case analytical subsets used for the principal models are illustrated in Figure 1, whereas variable availability across the dataset is summarized in Supplementary Table S1 and descriptive baseline characteristics are presented in Table 1 and Supplementary Table S2.

### 3.2. Baseline Characteristics According to Elevated LVEDP

Patients were stratified according to the primary outcome as LVEDP < 15 mmHg and LVEDP ≥ 15 mmHg. Compared with patients without elevated LVEDP, those with LVEDP ≥ 15 mmHg had significantly higher serum creatinine levels (*p* = 0.026) and higher invasive dPAP values (*p* = 0.043). As expected, PCWP, available in a smaller subgroup, was higher among patients with elevated LVEDP (*p* = 0.004). This finding was considered supportive and was not used as a substitute for direct LVEDP measurement. The supportive relationship between PCWP and directly measured LVEDP in the PCWP-available subgroup is shown in Supplementary Figure S3. In contrast, age, hemoglobin, LVEF, echocardiographic sPAP, TR Vmax, invasive sPAP, and mPAP did not differ significantly between LVEDP strata. Among patients with paired LVEDP and dPAP measurements, the dPAP−LVEDP gradient was significantly lower in patients with elevated LVEDP than in those without elevated LVEDP (1.0 [−10.0 to 5.0] vs. 12.0 [4.8 to 15.2] mmHg; *p* < 0.001). In an exploratory analysis, LVEDP exceeded dPAP in 23 of 72 patients (31.9%). This subgroup was characterized primarily by markedly higher LVEDP rather than by higher dPAP or echo-sPAP, suggesting potential LVEDP–dPAP dissociation in a subset of patients. Detailed exploratory comparisons are provided in Supplementary Table S10. Detailed comparisons of continuous baseline, laboratory, echocardiographic, and invasive hemodynamic variables according to LVEDP group are shown in Table 1. Exploratory derived hemodynamic indices, including PA pulse pressure and the dPAP−LVEDP gradient, are summarized separately in Supplementary Table S6. These indices were used only descriptively to illustrate relative pulmonary-to-left ventricular pressure relationships and were not treated as validated diagnostic markers or incorporated into the principal models.

### 3.3. Clinical Characteristics According to Elevated LVEDP

Sex distribution did not differ significantly between the two LVEDP groups (*p* = 0.576). Among the tested clinical comorbidities and categorical baseline characteristics, none showed a significant association with elevated LVEDP. Categorical clinical characteristics according to LVEDP group are summarized in Supplementary Table S2.

### 3.4. Noninvasive Markers, Invasive Pulmonary Hemodynamics, and Directly Measured LVEDP

**Figure 2:**
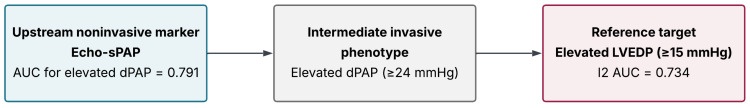
Conceptual relationship between noninvasive pulmonary pressure estimates, invasive dPAP, and directly measured LVEDP. Conceptual schematic of the study framework. Echocardiographic systolic pulmonary artery pressure (echo-sPAP) represented the principal upstream noninvasive marker, elevated invasive pulmonary artery diastolic pressure (dPAP ≥ 24 mmHg) represented the intermediate invasive pulmonary hemodynamic phenotype, and elevated directly measured LVEDP (LVEDP ≥ 15 mmHg) represented the reference target. This schematic summarizes the analytical structure linking noninvasive assessment, invasive pulmonary hemodynamics, and directly measured left ventricular filling pressure.

The analyses were structured to evaluate the relationship between noninvasive markers, invasive pulmonary hemodynamics, and directly measured LVEDP. In the noninvasive-to-invasive bridge layer, echo-sPAP emerged as the most informative upstream noninvasive marker. In the invasive-to-LVEDP layer, dPAP represented the most consistent invasive pulmonary hemodynamic correlate of elevated LV filling pressure. This structure was supported by both the group comparisons and the model-based analyses. The overall study framework is illustrated conceptually in Figure 2. Correlation analyses among the principal noninvasive, invasive, and LVEDP variables are summarized in Supplementary Table S5.

### 3.5. Bridge Model for Elevated dPAP

To determine whether noninvasive markers reflected an invasive pulmonary hemodynamic phenotype, a bridge model was constructed using elevated dPAP (≥24 mmHg) as the outcome. In this model, echo-sPAP alone showed good discriminatory ability, with an AUC of 0.791 (95% CI 0.695–0.888). This finding supports the role of echo-sPAP as an upstream noninvasive marker of invasive pulmonary hemodynamic burden. Bootstrap internal validation showed minimal optimism, with an optimism-corrected Dxy of 0.588, corresponding to an optimism-corrected AUC of approximately 0.794. The optimism-corrected calibration slope was 1.028, supporting the internal stability of the association between echo-sPAP and elevated dPAP. Regression estimates and internal validation metrics for the bridge model are summarized in Table 2 and Table 3. The ROC curve for this model is shown in Supplementary Figure S4, and the continuous association between echo-sPAP and invasive dPAP is illustrated in Supplementary Figure S1.

### 3.6. Noninvasive Model for Elevated LVEDP

A noninvasive model combining echo-sPAP and serum creatinine was then evaluated for elevated LVEDP. In the multivariable logistic regression model, echo-sPAP did not remain independently significant (β = 0.016, *p* = 0.296), whereas serum creatinine showed borderline statistical significance (β = 2.019, *p* = 0.056). The odds ratio for serum creatinine was 7.53 (95% CI 0.95–59.95), while the odds ratio for echo-sPAP was 1.02 per mmHg (95% CI 0.99–1.05). Overall discrimination of this noninvasive model was modest, with an AUC of 0.664 (95% CI 0.522–0.806) and a Brier score of 0.198.

**Figure 3:**
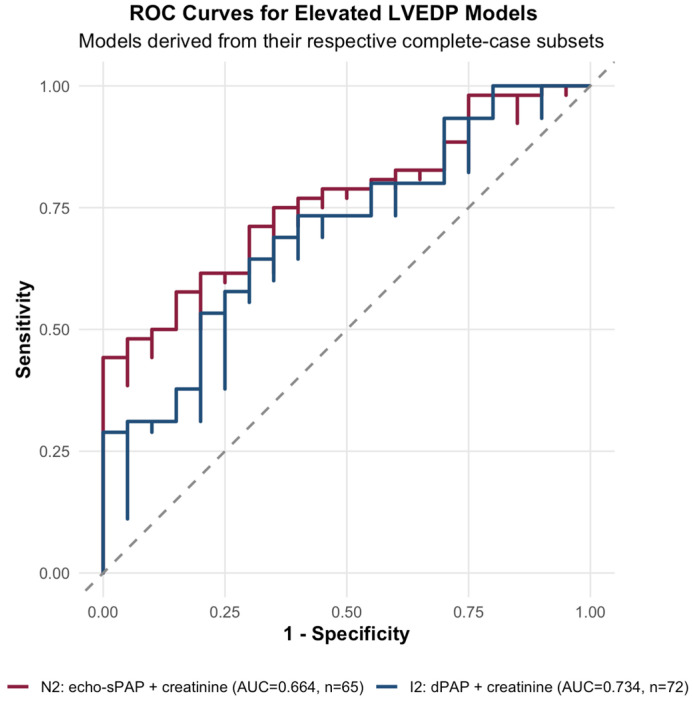
Receiver operating characteristic curves for the principal models of elevated LVEDP. Receiver operating characteristic curves comparing the principal noninvasive model (N2: echocardiographic systolic pulmonary artery pressure [echo-sPAP] plus serum creatinine) and the invasive validation model (I2: pulmonary artery diastolic pressure [dPAP] plus serum creatinine) for elevated directly measured LVEDP. Each curve was derived from its respective complete-case subset according to model-specific data availability.

**Figure 4:**
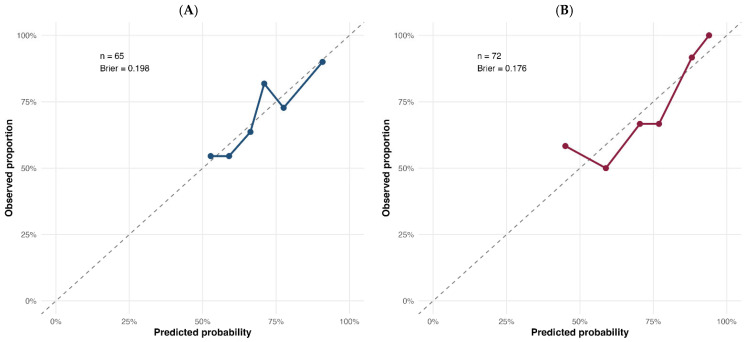
Calibration plots of the principal noninvasive and invasive validation models. Panel (**A**) shows the calibration of the principal noninvasive model (N2: echo-sPAP plus serum creatinine), and Panel (**B**) shows the calibration of the invasive validation model (I2: dPAP plus serum creatinine) for elevated directly measured LVEDP. The colored solid lines with circular markers represent the observed event proportions across predicted probability groups for each model. The grey dashed diagonal line represents perfect calibration, where predicted probabilities equal observed proportions. Sample sizes and Brier scores are displayed within the panels.

Bootstrap internal validation demonstrated limited but non-negligible optimism. The optimism-corrected Dxy was 0.249, corresponding to an optimism-corrected AUC of approximately 0.624. The optimism-corrected calibration slope was 0.824. Calibration performance was acceptable, with a mean absolute calibration error of 0.027 in the bootstrap calibration plot. The regression coefficients, discrimination metrics, and internal validation results of the noninvasive model are summarized in Table 2 and Table 3; the corresponding ROC and calibration plots are shown in Figure 3 and Figure 4, respectively.

### 3.7. Invasive Validation Model for Elevated LVEDP

An invasive validation model combining dPAP and serum creatinine was then evaluated for elevated LVEDP. Both variables remained independently associated with elevated LVEDP in multivariable analysis. For dPAP, the regression coefficient was 0.082 (*p* = 0.021), corresponding to an odds ratio of 1.085 per mmHg increase (95% CI 1.02–1.17). For serum creatinine, the regression coefficient was 2.193 (*p* = 0.041), corresponding to an odds ratio of 8.96 (95% CI 1.38–98.31).

This invasive validation model showed the highest discriminatory performance among the principal LVEDP models, with an AUC of 0.734 (95% CI 0.617–0.850) and a Brier score of 0.176. In bootstrap internal validation, the optimism-corrected Dxy was 0.423, corresponding to an optimism-corrected AUC of approximately 0.711. The optimism-corrected calibration slope was 0.912, indicating better internal stability than the noninvasive model. The bootstrap calibration plot showed a mean absolute calibration error of 0.061. The invasive validation model estimates and performance metrics are summarized in Table 2 and Table 3; the corresponding ROC and calibration plots are shown in Figure 3 and Figure 4. The continuous association between dPAP and directly measured LVEDP is illustrated in Supplementary Figure S2.

### 3.8. Sensitivity, Threshold, and Exploratory Analyses

Direct comparison of the noninvasive model (echo-sPAP + creatinine) and the invasive validation model (dPAP + creatinine) showed numerically better discrimination for the invasive model. However, DeLong comparison did not demonstrate a statistically significant difference between the two ROC curves (AUC 0.664 vs. 0.734, *p* = 0.459). Thus, although the invasive validation model performed better in absolute terms, the difference could not be confirmed statistically in this sample. Direct visual comparison of the two principal LVEDP models is provided in Figure 3, and their discrimination and internal validation metrics are summarized in Table 3.

In eGFR-based sensitivity analyses, the principal findings were largely preserved. Median eGFR was lower in patients with elevated LVEDP than in those without elevated LVEDP, although this difference did not reach conventional statistical significance (80.7 [61.4–103.0] vs. 99.0 [78.2–109.0] mL/min/1.73 m^2^; *p* = 0.056). Replacing creatinine with eGFR yielded similar performance for the invasive validation model (dPAP + eGFR: AUC 0.731, 95% CI 0.613–0.849) compared with the creatinine-based model. The bridge model also remained stable when eGFR was included (echo-sPAP + eGFR: AUC 0.796, 95% CI 0.700–0.893). The noninvasive eGFR-based LVEDP model showed lower discrimination (AUC 0.620, 95% CI 0.480–0.760). These analyses are summarized in Supplementary Table S8.

To address the potential influence of temporal separation between echocardiographic and catheterization assessments, we performed a sensitivity analysis restricted to patients whose examinations were completed within 7 days (Supplementary Table S7). In this subgroup, the principal findings were directionally preserved. The bridge model using echo-sPAP for elevated dPAP showed an AUC of 0.899 (95% CI, 0.784–1.000; n = 26). The noninvasive model for elevated LVEDP yielded an AUC of 0.722 (95% CI, 0.498–0.946; n = 24), whereas the invasive validation model yielded an AUC of 0.824 (95% CI, 0.658–0.990; n = 24). Given the smaller sample size, these analyses were interpreted as supportive.

Multiple imputation sensitivity analyses yielded directionally consistent results for the principal models. Echo-sPAP remained associated with elevated dPAP, and dPAP remained associated with elevated LVEDP in the invasive validation model. The noninvasive model remained modest, with echo-sPAP remaining nonsignificant and creatinine showing borderline significance. These results are summarized in Supplementary Table S11.

ROC-derived optimal cut-off values are presented in Supplementary Table S9. For identifying elevated dPAP, echo-sPAP showed an optimal threshold of 46 mmHg, with a sensitivity of 64.4% and specificity of 77.8%. For identifying elevated LVEDP, dPAP had an optimal threshold of 27.5 mmHg, showing high specificity (90.0%) but limited sensitivity (38.5%). In the PCWP-available subgroup, a PCWP threshold of 14.5 mmHg showed the strongest threshold-based performance for elevated LVEDP. Because elevated LVEDP was frequent in this selected catheterization cohort, PPV and NPV were considered exploratory.

Because some patients showed LVEDP values exceeding dPAP, we performed an exploratory analysis of the LVEDP–dPAP relationship. Among 72 patients with paired LVEDP and dPAP measurements, LVEDP exceeded dPAP in 23 patients (31.9%). These patients had markedly higher LVEDP (45.0 [26.0–54.5] vs. 15.0 [12.0–20.0] mmHg; *p* < 0.001), whereas dPAP did not differ significantly between groups. Invasive sPAP was lower in the LVEDP > dPAP group, while echo-sPAP and PCWP did not differ significantly. Detailed exploratory comparisons are presented in Supplementary Table S10.

### 3.9. Overall Interpretation of the Principal Findings

Overall, the findings were consistent with the prespecified analytical framework: echo-sPAP primarily identified the intermediate invasive dPAP phenotype, whereas dPAP combined with creatinine provided the strongest signal for elevated directly measured LVEDP. Additional univariable and sensitivity analyses are summarized in Supplementary Tables S3 and S4.

## 4. Discussion

In this retrospective routine catheterization cohort, we identified a stepwise relationship between noninvasive echocardiographic findings, invasive pulmonary hemodynamics, and directly measured left ventricular end-diastolic pressure. The main findings were threefold. First, echocardiographic systolic pulmonary artery pressure showed good discriminatory performance for an invasive pulmonary hemodynamic phenotype defined by elevated dPAP. Second, invasive dPAP, particularly when combined with serum creatinine, provided the most informative signal for elevated directly measured LVEDP. Third, a fully noninvasive model based on echo-sPAP and creatinine showed only modest discrimination for elevated LVEDP. Taken together, these findings support a stepwise hemodynamic relationship rather than a simple direct association between echocardiography and left ventricular filling pressure.

A key implication of these findings is that echocardiographic pulmonary pressure estimates may not primarily function as direct stand-alone surrogates of LVEDP, but rather as upstream markers of an intermediate invasive pulmonary hemodynamic burden. In our cohort, echo-sPAP performed substantially better for elevated dPAP than for elevated LVEDP itself. This pattern is physiologically plausible. Elevated left-sided filling pressure may be transmitted backward to the pulmonary circulation, where it becomes more closely reflected in pulmonary artery diastolic pressure [13]. Within this framework, dPAP appears to represent a more proximal invasive expression of left-sided hemodynamic burden, whereas echocardiographic pulmonary pressure estimates remain upstream, indirect markers [14]. This interpretation may help explain why conventional noninvasive filling-pressure markers often show only modest agreement with direct invasive measurements when evaluated in isolation.

The comparative model performance further supports this interpretation. The noninvasive model combining echo-sPAP and creatinine yielded only modest discrimination for elevated LVEDP, whereas the invasive validation model incorporating dPAP and creatinine showed better apparent discrimination, a lower Brier score, and more favorable bootstrap-corrected performance. Although the difference in AUC did not reach statistical significance in DeLong comparison, the overall pattern consistently favored the invasive model. Accordingly, in this cohort, the hemodynamic signal most relevant to elevated LVEDP appeared to be captured more effectively at the level of invasive pulmonary hemodynamics than at the purely noninvasive level.

Among the invasive variables, dPAP emerged as the most informative bridging parameter. This was supported by its significant difference across LVEDP strata and by its independent association with elevated LVEDP in multivariable analysis. In contrast, invasive sPAP and mPAP did not show the same degree of discriminatory value in the primary analyses. This pattern suggests that dPAP may more closely reflect the pulmonary vascular consequence of elevated downstream filling pressure in this routine catheterization population. The PCWP subgroup analysis was directionally supportive, although the smaller sample size of that subgroup precludes firm conclusions. This finding should be interpreted as an expected supportive observation rather than as a central novel result, because PCWP and LVEDP are physiologically related but may diverge under selected clinical and technical conditions.

The LVEDP–dPAP relationship also deserves specific comment. In patients with elevated LVEDP, the dPAP−LVEDP gradient was significantly lower than in those without elevated LVEDP, and LVEDP exceeded dPAP in approximately one-third of patients with paired measurements. This finding should not be interpreted solely as measurement error. Rather, it may reflect potential dissociation between instantaneous left ventricular diastolic pressure and pulmonary artery diastolic pressure in routine catheterization recordings. Several factors may contribute to this pattern, including non-simultaneous pressure acquisition, respiratory phase, acute loading conditions, ventricular stiffness, valvular disease, pulmonary vascular compliance, and routine measurement variability. In our exploratory comparison, this subgroup was characterized primarily by markedly higher LVEDP rather than by a clear difference in echo-sPAP or baseline comorbidities. Importantly, this observation reinforces that dPAP should be interpreted as an intermediate pulmonary hemodynamic marker rather than as a direct one-to-one surrogate of LVEDP.

The threshold analyses further support the stepwise interpretation of the findings. Echo-sPAP showed its most informative threshold-based performance for elevated dPAP rather than for elevated LVEDP. Conversely, dPAP showed high specificity but limited sensitivity for elevated LVEDP, suggesting that it may be more useful as a rule-in marker than as a rule-out marker in this selected catheterization cohort.

Creatinine was the most consistent clinical support variable across models. However, it should not be interpreted as a direct surrogate of LV filling pressure. Rather, its contribution likely reflects the broader cardiorenal and hemodynamic vulnerability within which elevated LVEDP occurs in real-world catheterization patients [15,16]. In this setting, higher filling pressures are often embedded within a more complex physiological milieu that includes congestion, impaired renal perfusion, chronic comorbidity burden, and reduced reserve [15]. From this perspective, creatinine may act as a contextual marker of systemic disease severity that complements the hemodynamic signal carried by dPAP, rather than serving as a mechanistic determinant of LVEDP itself [17]. Because serum creatinine may incompletely represent renal function, we repeated the principal models using CKD-EPI 2021 eGFR. These analyses yielded directionally consistent results, particularly for the invasive validation model, in which dPAP remained associated with elevated LVEDP after adjustment for eGFR. The noninvasive eGFR-based model showed weaker discrimination, whereas the bridge model remained essentially unchanged. These findings suggest that the renal-function signal observed in the primary analysis was not solely dependent on the use of serum creatinine, although the wide confidence intervals and modest sample size warrant cautious interpretation.

An important conceptual point is that this study should not be interpreted as a simple re-demonstration that echocardiography relates to filling pressure. That general principle is already recognized. The novelty of the present work lies in the layered integration of three components within the same cohort: routinely available noninvasive markers, invasive pulmonary artery hemodynamics, and directly measured LVEDP. In particular, our results support the concept of invasive dPAP as an intermediate hemodynamic phenotype linking upstream noninvasive findings to directly measured elevated left ventricular filling pressure [3]. In this design, the invasive component serves as a mechanistic anchor supporting the physiological coherence of the noninvasive signal rather than functioning as a post-catheterization bedside score.

The proposed framework is therefore complementary to, rather than a replacement for, guideline-based diastolic assessment. ASE/EACVI algorithms remain the standard noninvasive approach for estimating LV filling pressure, particularly when complete Doppler and structural parameters are available. The present study addresses a different question: whether routinely available pulmonary pressure estimates and laboratory markers can be physiologically linked to directly measured LVEDP through an intermediate invasive pulmonary hemodynamic phenotype. Thus, the incremental value of the proposed framework lies in its mechanistic integration of echo-sPAP, invasive dPAP, and direct LVEDP within the same routine catheterization cohort, rather than in replacing established echocardiographic algorithms.

From a clinical standpoint, the value of the present study lies not in proposing a definitive noninvasive substitute for catheter-based hemodynamic assessment, but in clarifying how routinely available findings may be interpreted within a physiologically anchored context. In practice, elevated echocardiographic pulmonary pressure estimates are often encountered in patients whose conventional diastolic assessment is incomplete, equivocal, or discordant with the overall clinical picture [1,3]. Our findings suggest that, in such settings, increased echo-sPAP, particularly when accompanied by impaired renal function, may identify a subgroup more likely to harbor an invasive pulmonary hemodynamic profile associated with elevated directly measured LVEDP. Accordingly, the principal clinical contribution of this study is not a stand-alone prediction rule, but a physiologically anchored framework for hemodynamic triage and risk enrichment. The noninvasive model should therefore be viewed as supportive rather than definitive.

This study has several strengths. Directly measured LVEDP was used as the primary invasive reference target rather than relying solely on PCWP-based surrogacy. In addition, the study integrated noninvasive markers, invasive pulmonary hemodynamics, and direct LVEDP within the same real-world cohort, and bootstrap internal validation was performed to provide a more realistic assessment of model stability.

Several limitations should also be acknowledged. This was a retrospective single-center study with a modest sample size, limiting generalizability and model complexity. In addition, the high prevalence of elevated LVEDP in this selected catheterization cohort may limit generalizability to broader populations with a lower pretest probability of elevated filling pressure. Not all echocardiographic variables were available in all patients, PCWP was assessed only in a subgroup, and temporal alignment between echocardiography and catheterization was not perfectly uniform. In addition, echo-sPAP was extracted from routine echocardiography reports rather than independently recalculated from raw Doppler and inferior vena cava measurements; therefore, variability in right atrial pressure estimation may have influenced the noninvasive pulmonary pressure estimates. Although echocardiography and catheterization were restricted to a 1-month interval, the measurements were not obtained simultaneously, and interval changes in clinical or hemodynamic status may still have attenuated some noninvasive-to-invasive associations. Importantly, when the analysis was restricted to patients with echocardiography and catheterization performed within 7 days, the direction of the principal associations was preserved. Nevertheless, this subgroup was small, and the resulting estimates were less precise; therefore, these findings should be viewed as supportive rather than definitive. Variable-specific missingness resulted in different complete-case cohorts across the descriptive and model-based analyses. Because missingness reflected the retrospective availability of routinely collected data rather than a prespecified research protocol, it cannot be assumed to be completely random. Selection bias therefore cannot be excluded, and the reported associations and model performances should be interpreted cautiously. Although complete-case analysis was retained as the primary approach because of variable-specific and partly structural missingness, multiple imputation sensitivity analyses were performed for the principal model variables with moderate missingness and yielded directionally consistent results. Variables with substantial nonavailability, such as PCWP and TR Vmax, were not imputed. LVEDP exceeded dPAP in a subset of patients, underscoring that dPAP should not be regarded as a direct one-to-one surrogate of LVEDP; this may reflect physiological dissociation, respiratory or loading conditions, non-simultaneous pressure acquisition, or routine measurement variability. Finally, the study was designed to develop an interpretable and physiologically grounded framework rather than a definitive clinical prediction tool. No prospective validation or prospectively adjudicated diagnostic accuracy data were available; therefore, the observed model performance should be interpreted as retrospective and internally validated only, and external validation in larger cohorts will be required before broader application can be considered.

## 5. Conclusions

In conclusion, routinely available noninvasive echocardiographic and laboratory findings appear to relate to directly measured left ventricular filling pressure through an intermediate invasive pulmonary hemodynamic pattern. Echo-sPAP showed its strongest signal at the level of elevated dPAP, whereas dPAP combined with creatinine provided the most informative model for elevated directly measured LVEDP. These findings support a hypothesis-generating hemodynamic framework linking noninvasive assessment to directly measured filling pressure and suggest a potential supportive role for noninvasive hemodynamic triage and physiological risk enrichment in selected clinical settings.

## Figures and Tables

**Table 1:** Baseline clinical, laboratory, echocardiographic, and invasive hemodynamic characteristics according to directly measured LVEDP.

Variable	LVEDP < 15 mmHg (*n* = 20)	LVEDP ≥ 15 mmHg (*n* = 55)	*p*-Value
**Age, years**	54.7 [49.3–67.8]	55.9 [48.1–64.4]	0.981
**Creatinine, mg/dL**	0.78 [0.60–1.02]	0.90 [0.80–1.23]	0.026
**Hemoglobin, g/dL**	13.1 [11.3–14.3]	12.2 [10.0–13.7]	0.149
**LVEF, %**	57.5 [22.8–60.0]	50.0 [25.0–60.0]	0.941
**Echo-sPAP, mmHg**	40.0 [35.0–57.8]	45.0 [40.0–60.0]	0.312
**TR Vmax, m/s †**	2.65 [2.10–3.20]	2.90 [2.63–3.18]	0.656
**dPAP, mmHg**	20.5 [15.5–25.0]	24.0 [19.8–31.0]	0.043
**sPAP, mmHg**	38.5 [27.8–63.0]	50.0 [38.0–58.0]	0.295
**mPAP, mmHg**	27.0 [19.8–42.1]	34.2 [24.9–40.2]	0.189
**PCWP, mmHg ‡**	13.0 [10.0–18.0]	25.0 [19.0–28.5]	0.004

**Abbreviations:** PCWP, pulmonary capillary wedge pressure; LVEDP, left ventricular end-diastolic pressure; dPAP, pulmonary artery diastolic pressure; sPAP, pulmonary artery systolic pressure; mPAP, mean pulmonary artery pressure; LVEF, left ventricular ejection fraction. Data are presented as median [interquartile range]. Continuous variables were compared using the Mann–Whitney U test. Elevated LVEDP was defined as LVEDP ≥ 15 mmHg. mPAP indicates mean pulmonary artery pressure calculated from recorded sPAP and dPAP values. Echo-sPAP indicates echocardiographically estimated systolic pulmonary artery pressure. † Available in 36 patients. ‡ Available in 37 patients.

**Table 2:** Multivariable logistic regression models for elevated LVEDP and the intermediate invasive pulmonary hemodynamic phenotype.

**(A) Principal Models for Elevated LVEDP**				
**Model**	**Variable**	**OR**	**95% CI**	***p*** **-Value**
**N2: Noninvasive model**	Echo-sPAP	1.017	0.986–1.048	0.296
**Creatinine, mg/dL**		7.53	0.95–59.95	0.056
**I2: Invasive validation model**	dPAP, mmHg	1.085	1.012–1.163	0.021
**Creatinine, mg/dL**		8.96	1.38–98.31	0.041
**(B) Bridge model for elevated dPAP**				
**Model**	**Variable**	**OR**	**95% CI**	***p*** **-value**
**B1: Bridge model**	Echo-sPAP, mmHg	1.092	1.044–1.142	<0.001

Abbreviations: LVEDP, left ventricular end-diastolic pressure; dPAP, pulmonary artery diastolic pressure; OR, odds ratio; CI, confidence interval. Elevated LVEDP was defined as LVEDP ≥ 15 mmHg. Elevated dPAP was defined as dPAP ≥ 24 mmHg. Odds ratios are shown with 95% confidence intervals. The noninvasive model evaluated clinically accessible variables before invasive confirmation; the invasive validation model assessed the hemodynamic association of dPAP with directly measured LVEDP; the bridge model assessed whether echo-sPAP identified an intermediate invasive pulmonary hemodynamic phenotype.

**Table 3:** Discrimination, calibration, and bootstrap internal validation of the principal models.

Model	Outcome	*n*	AUC	95% CI	Brier Score	Optimism-Corrected AUC *	Calibration Slope *
**B1**	High dPAP	81	0.791	0.695–0.888	0.185	0.794	1.028
**N2**	High LVEDP	65	0.664	0.522–0.806	0.198	0.624	0.824
**I2**	High LVEDP	72	0.734	0.617–0.850	0.176	0.711	0.912

**Additional comparison:** DeLong *p* value for N2 vs. I2 = 0.459. **Abbreviations:** AUC, area under the curve; LVEDP, left ventricular end-diastolic pressure; dPAP, pulmonary artery diastolic pressure. AUC indicates area under the receiver operating characteristic curve. Brier score was used as a global measure of predictive accuracy, with lower values indicating better overall performance. Internal validation was performed using bootstrap resampling (1000 repetitions). * Optimism-corrected AUC values were derived from the bootstrap-corrected Somers’ Dxy statistic; calibration slope values closer to 1.0 indicate better internal stability.

## Data Availability

The data underlying this study are available from the corresponding author upon reasonable request, subject to institutional and privacy restrictions.

## Supplementary Materials

The following supporting information can be downloaded at: https://www.mdpi.com/article/10.3390/diagnostics16101559/s1, Figure S1: Scatter plot of echocardiographic systolic pulmonary artery pressure versus invasive pulmonary artery diastolic pressure; Figure S2: Scatter plot of invasive pulmonary artery diastolic pressure versus directly measured LVEDP; Figure S3: Scatter plot of PCWP versus directly measured LVEDP; Figure S4: Receiver operating characteristic curve for the bridge model of elevated dPAP; Table S1: Variable availability across the study dataset; Table S2: Categorical clinical characteristics according to directly measured LVEDP; Table S3: Univariable discrimination of principal invasive and noninvasive markers; Table S4: Sensitivity analyses of alternative noninvasive and bridge models; Table S5: Spearman correlations among noninvasive markers, invasive pulmonary hemodynamics, and directly measured LVEDP; Table S6: Exploratory derived hemodynamic indices according to directly measured LVEDP; Table S7: Sensitivity analysis restricted to patients with echocardiography and catheterization within 7 days; Table S8: eGFR-based sensitivity analyses; Table S9: ROC-derived optimal cut-off values and diagnostic indices; Table S10: Exploratory comparison according to LVEDP–dPAP relationship; Table S11: Multiple imputation sensitivity analyses for the principal logistic regression models; Table S12: Conceptual comparison of approaches for estimating or interpreting elevated left ventricular filling pressure.
