# Supplementary material for: A Hemodynamic Bridge from Echocardiography to Directly Measured Left Ventricular End-Diastolic Pressure: The Intermediate Role of Pulmonary Artery Diastolic Pressure in a Routine Catheterization Cohort
Source: Diagnostics (Basel). 2026 May 20;16(10):1559. doi: 10.3390/diagnostics16101559 (PMC13205698; doi:10.3390/diagnostics16101559)
Supplement: Supplementary file 1 [file diagnostics-16-01559-s001.zip › diagnostics-4284419-supplementary.pdf]

Supplementary Table S1. Variable availability across the study dataset.

Variable	Available n	Missing n
**Direct LVEDP**	75	36
**dPAP**	94	17
**sPAP**	95	16
**mPAP**	94	17
**PCWP**	37	74
**Echo-sPAP**	83	28
**TR Vmax**	36	75
**Creatinine**	97	14
**Hemoglobin**	97	14

Supplementary Table S2. Categorical clinical characteristics according to directly measured LVEDP.

Variable	LVEDP <15 mmHg	LVEDP ≥15 mmHg	p-value
**Male sex, n/N (%)**	12/20 (60.0%)	29/55 (52.7%)	0.576
**Hypertension, n/N (%)**	5/18 (27.8%)	21/54 (38.9%)	0.571
**Diabetes mellitus, n/N (%)**	5/18 (27.8%)	15/54 (27.8%)	1.000
**Coronary artery disease, n/N (%)**	6/19 (31.6%)	19/54 (35.2%)	0.777
**Atrial fibrillation history, n/N (%)**	6/19 (31.6%)	15/53 (28.3%)	1.000
**COPD, n/N (%)**	1/18 (5.6%)	3/54 (5.6%)	1.000
**CKD, n/N (%)**	0/18 (0.0%)	3/54 (5.6%)	0.568
**Valvular disease, n/N (%)**	12/19 (63.2%)	34/54 (63.0%)	1.000

Table footnote: Data are presented as n/N (%). Denominators vary across variables because of variable-specific missing data. P-values were derived from chi-square or Fisher's exact test, as appropriate.

Supplementary Table S3. Univariable discrimination of principal invasive and noninvasive markers.

Predictor	Outcome	n	AUC	95% CI
**Echo-sPAP**	High LVEDP	65	0.579	0.413–0.746
**dPAP**	High LVEDP	72	0.655	0.522–0.788
**Echo-sPAP**	High dPAP	81	0.791	0.695–0.888
**PCWP***	High LVEDP	29	0.839	0.659–1.000

Table footnote: This table summarizes univariable discrimination for the main predictors. Area under the receiver operating characteristic curve (AUC) values are presented with 95% confidence intervals. PCWP is shown as a supportive subgroup analysis only.

*PCWP available only in a limited subset.

Supplementary Table S4. Sensitivity analyses of alternative noninvasive and bridge models.

Model	Outcome	n	Variable	OR	95% CI	p-value	AUC
**N1**	High LVEDP	65	Echo-sPAP	1.008	0.978–1.038	0.615	0.579
**N3**	High LVEDP	65	Echo-sPAP	1.011	0.979–1.044	0.501	0.687
**Creatinine, mg/dL**				7.06	0.86–58.00	0.069	

**Hemoglobin, g/dL**				0.83	0.64–1.07	0.156	
**I1**	High LVEDP	72	dPAP, mmHg	1.075	1.004–1.152	0.039	0.655
**B2**	High dPAP	81	Echo-sPAP, mmHg	1.092	1.044–1.142	<0.001	0.798
**Creatinine, mg/dL**				1.01	0.65–1.57	0.977	
**B3**	High dPAP	36	TR Vmax, m/s	21.040	2.540–174.040	0.0047	0.856
**Creatinine, mg/dL**				0.63	0.10–4.01	0.626	

Table footnote: Sensitivity analyses were performed to assess model robustness. TR Vmax-based models were limited by smaller sample size and should be interpreted as supportive rather than primary evidence.

Supplementary Table S5. Spearman correlations among noninvasive markers, invasive pulmonary hemodynamics, and directly measured LVEDP.

Variable pair	n	Spearman rho	p-value
**Echo-sPAP vs. dPAP**	81	0.509	<0.001
**TR Vmax vs. dPAP**	36	0.572	<0.001
**dPAP vs. LVEDP**	72	0.267	0.023
**Echo-sPAP vs. LVEDP**	65	0.132	0.294
**PCWP vs. LVEDP**	29	0.633	<0.001
**Creatinine vs. LVEDP**	75	0.138	0.237
**Hemoglobin vs. LVEDP**	75	-0.149	0.203
**LVEF vs. LVEDP**	75	-0.152	0.193
**sPAP vs. LVEDP**	73	0.043	0.716
**mPAP vs. LVEDP**	72	0.122	0.307

Table footnote: Spearman correlation coefficients are reported because of the non-normal distribution expected for several hemodynamic variables. The strongest correlations in the proposed bridge framework were observed for echo-sPAP vs. dPAP and PCWP vs. LVEDP.

Supplementary Table S6. Exploratory derived hemodynamic indices according to directly measured LVEDP.

Variable	LVEDP <15 mmHg	LVEDP ≥15 mmHg	p-value
**PA pulse pressure, mmHg**	19.5 [11.8–31.0]	20.0 [15.0–28.0]	0.960
**dPAP − LVEDP**	12.0 [4.8–15.2]	1.0 [-10.0–5.0]	<0.001

Table footnote: Data are presented as median [interquartile range]. P-values were derived from the Mann–Whitney U test. These variables were treated as exploratory descriptive indices and were not incorporated into the principal multivariable models. The dPAP−LVEDP gradient was used to describe relative pressure dissociation between pulmonary artery diastolic pressure and directly measured LVEDP, rather than as a validated diagnostic marker.

Supplementary Table S7. Sensitivity analysis restricted to patients with echocardiography and catheterization within 7 days.

Model	Outcome	Main analysis n	Main AUC (95% CI)	≤7-day n	≤7-day AUC (95% CI)
**B1: echo-sPAP**	High dPAP	81	0.791 (0.695–0.888)	26	0.899 (0.784–1.000)

**N2: echo-sPAP + creatinine**	High LVEDP	65	0.664 (0.522–0.806)	24	0.722 (0.498–0.946)
**I2: dPAP + creatinine**	High LVEDP	72	0.734 (0.617–0.850)	24	0.824 (0.658–0.990)

Table footnote: The ≤7-day sensitivity analysis was performed in patients whose echocardiographic and catheterization assessments were completed within 7 days. AUC values are shown with 95% confidence intervals. Because of the reduced sample size, these analyses were considered supportive rather than confirmatory.

Supplementary Table S8. eGFR-based sensitivity analyses.

Model	Outcome	n	Variables	OR	95% CI	p-value	AUC
**N2-eGFR**	High LVEDP	65	echo-sPAP	1.013	0.985–1.046	0.382	0.620
**eGFR**				0.980	0.956–1.002	0.092	
**I2-eGFR**	High LVEDP	72	dPAP	1.078	1.015–1.161	0.028	0.731
**eGFR**				0.979	0.954–1.001	0.075	
**B2-eGFR**	High dPAP	81	echo-sPAP	1.092	1.048–1.148	<0.001	0.796
**eGFR**				0.998	0.980–1.017	0.827	

Table footnote: eGFR was calculated using the 2021 CKD-EPI creatinine equation. Odds ratios are presented per 1-unit increase for continuous variables. These analyses were performed as sensitivity analyses in response to concerns that eGFR may better represent renal function than serum creatinine.

Supplementary Table S9. ROC-derived optimal cut-off values and diagnostic indices.

Predictor/model	Outcome	n	Events	AUC (95% CI)	Youden threshold	Sensitivity	Specificity	PPV	NPV
**Echo-sPAP for high dPAP**	High dPAP	81	45	0.791 (0.695–0.888)	46.0	0.644	0.778	0.784	0.636
**Echo-sPAP for high LVEDP**	High LVEDP	65	45	0.579 (0.413–0.746)	42.5	0.689	0.550	0.775	0.440
**dPAP for high LVEDP**	High LVEDP	72	52	0.655 (0.522–0.788)	27.5	0.385	0.900	0.909	0.360
**PCWP for high LVEDP**	High LVEDP	29	20	0.839 (0.659–1.000)	14.5	0.950	0.667	0.864	0.857
**N2 model for high LVEDP**	High LVEDP	65	45	0.664 (0.522–0.806)	0.666	0.644	0.650	0.806	0.448

**I2 model for high LVEDP**	High LVEDP	72	52	0.734 (0.617–0.850)	0.817	0.442	0.950	0.958	0.396

Table footnote: ROC-derived thresholds were identified using the Youden index. High LVEDP was defined as LVEDP ≥15 mmHg, and high dPAP as dPAP ≥24 mmHg. PPV and NPV should be interpreted cautiously because elevated LVEDP was frequent in this selected catheterization cohort. Model probability thresholds are exploratory and are not intended as definitive clinical decision cut-offs.

Supplementary Table S10. Exploratory comparison according to LVEDP–dPAP relationship.

Variable	LVEDP ≤ dPAP (n=49)	LVEDP > dPAP (n=23)	p-value
**Age, years**	53.4 [47.4–64.2]	61.0 [52.8–69.5]	0.106
**LVEDP, mmHg**	15.0 [12.0–20.0]	45.0 [26.0–54.5]	<0.001
**dPAP, mmHg**	24.0 [20.0–30.0]	20.0 [14.5–29.5]	0.140
**dPAP − LVEDP, mmHg**	7.0 [3.0–15.0]	−19.0 [−33.5 to −5.5]	<0.001
**sPAP, mmHg**	50.0 [37.0–60.0]	40.0 [26.0–52.5]	0.045
**mPAP, mmHg**	33.0 [26.0–43.0]	26.7 [18.7–37.7]	0.086
**Echo-sPAP, mmHg**	45.0 [40.0–63.0]	45.0 [38.5–55.0]	0.562
**PCWP, mmHg**	20.5 [14.2–25.0]	25.5 [19.0–26.8]	0.327
**LVEF, %**	50.0 [22.0–60.0]	53.0 [31.5–60.0]	0.566
**Creatinine, mg/dL**	0.9 [0.7–1.1]	0.9 [0.8–1.1]	0.524
**eGFR, mL/min/1.73 m²**	95.8 [71.1–107.8]	76.7 [63.6–101.1]	0.290
**AF history, n/N (%)**	11/47 (23.4%)	10/23 (43.5%)	0.149
**Valvular disease, n/N (%)**	29/48 (60.4%)	15/23 (65.2%)	0.898

Supplementary Table S11. Multiple imputation sensitivity analyses for the principal logistic regression models.

Model	Outcome	Variable	Pooled OR (95% CI)	p-value
**MI-N2: echo-sPAP + creatinine**	High LVEDP	echo-sPAP	1.014 (0.983–1.046)	0.360
**MI-N2: echo-sPAP + creatinine**	High LVEDP	Creatinine	8.482 (0.960–74.948)	0.054
**MI-I2: dPAP + creatinine**	High LVEDP	dPAP	1.085 (1.011–1.164)	0.024
**MI-I2: dPAP + creatinine**	High LVEDP	Creatinine	8.962 (1.053–76.249)	0.045
**MI-B1: echo-sPAP**	High dPAP	echo-sPAP	1.086 (1.040–1.135)	<0.001
**MI-B2: echo-sPAP + creatinine**	High dPAP	echo-sPAP	1.086 (1.040–1.135)	<0.001
**MI-B2: echo-sPAP + creatinine**	High dPAP	Creatinine	0.986 (0.625–1.556)	0.950

Table footnote: Multiple imputation by chained equations was performed using 20 imputed datasets. Outcomes were not imputed. Continuous variables were imputed using predictive mean matching, and binary variables were imputed using logistic regression. PCWP and TR Vmax were not imputed because of substantial or potentially structural nonavailability. Odds ratios are pooled estimates across imputed datasets.

Supplementary Table S12. Conceptual comparison of approaches for estimating or interpreting elevated left ventricular filling pressure.

Approach	Main variables	Reference/target	Strengths	Limitations	Role in the present study
**ASE/EACVI diastoli**	E/e′, septal/lateral e′, LA	Noninvasive estimation of LV filling	Standardized, guideline-supported,	May be indeterminate or discordant in	Provides the established noninvasive

**c function algorithm**	volume index, TR velocity, additional structural and Doppler parameters	pressure / diastolic dysfunction	clinically familiar, integrates multiple echocardiographic domains	real-world patients; requires complete Doppler and structural data; performance may vary across disease phenotypes	context against which the present physiologic framework is interpreted
**Echocardiographic pulmonary pressure markers**	Echo-sPAP, TR Vmax	Noninvasive estimate of pulmonary pressure burden	Widely available in routine echocardiography when TR signal is adequate; reflects downstream pulmonary hemodynamic consequences	Indirect marker; depends on TR envelope quality and right atrial pressure estimation; may not directly reflect LVEDP	Used as upstream noninvasive markers hypothesized to identify an invasive pulmonary hemodynamic phenotype
**PCWP-based invasive assessment**	PCWP / pulmonary artery wedge pressure	Surrogate of left atrial pressure and LV filling pressure	Invasive hemodynamic marker closely related to left-sided filling pressure; clinically established in right heart catheterization	Not always available; may diverge from LVEDP in selected conditions; affected by waveform quality, respiratory phase, atrial/mitral pathology	Evaluated as a supportive subgroup variable rather than the primary reference target
**Direct LVEDP measurement**	Left ventricular end-diastolic pressure	Direct invasive LV filling pressure	Direct catheter-based measurement; avoids reliance on PCWP surrogacy	Invasive; not routinely available outside catheterization; may vary with loading conditions and measurement technique	Used as the primary reference outcome
**Proposed dPAP bridge framework**	Echo-sPAP → invasive dPAP → directly measured LVEDP	Intermediate pulmonary hemodynamic pathway linking noninvasive	Integrates noninvasive findings, invasive pulmonary hemodynamics, and direct LVEDP within the same cohort;	Exploratory; not a replacement for guideline diastolic assessment or invasive measurement; requires external validation	Tests whether echo-sPAP relates to LVEDP through an intermediate invasive pulmonary hemodynamic phenotype

	markers to LVEDP	physiologically interpretable

Table footnote: ASE/EACVI indicates American Society of Echocardiography/European Association of Cardiovascular Imaging; LV, left ventricular; LVEDP, left ventricular end-diastolic pressure; LA, left atrial; TR, tricuspid regurgitation; PCWP, pulmonary capillary wedge pressure; echo-sPAP, echocardiographic systolic pulmonary artery pressure; dPAP, pulmonary artery diastolic pressure.

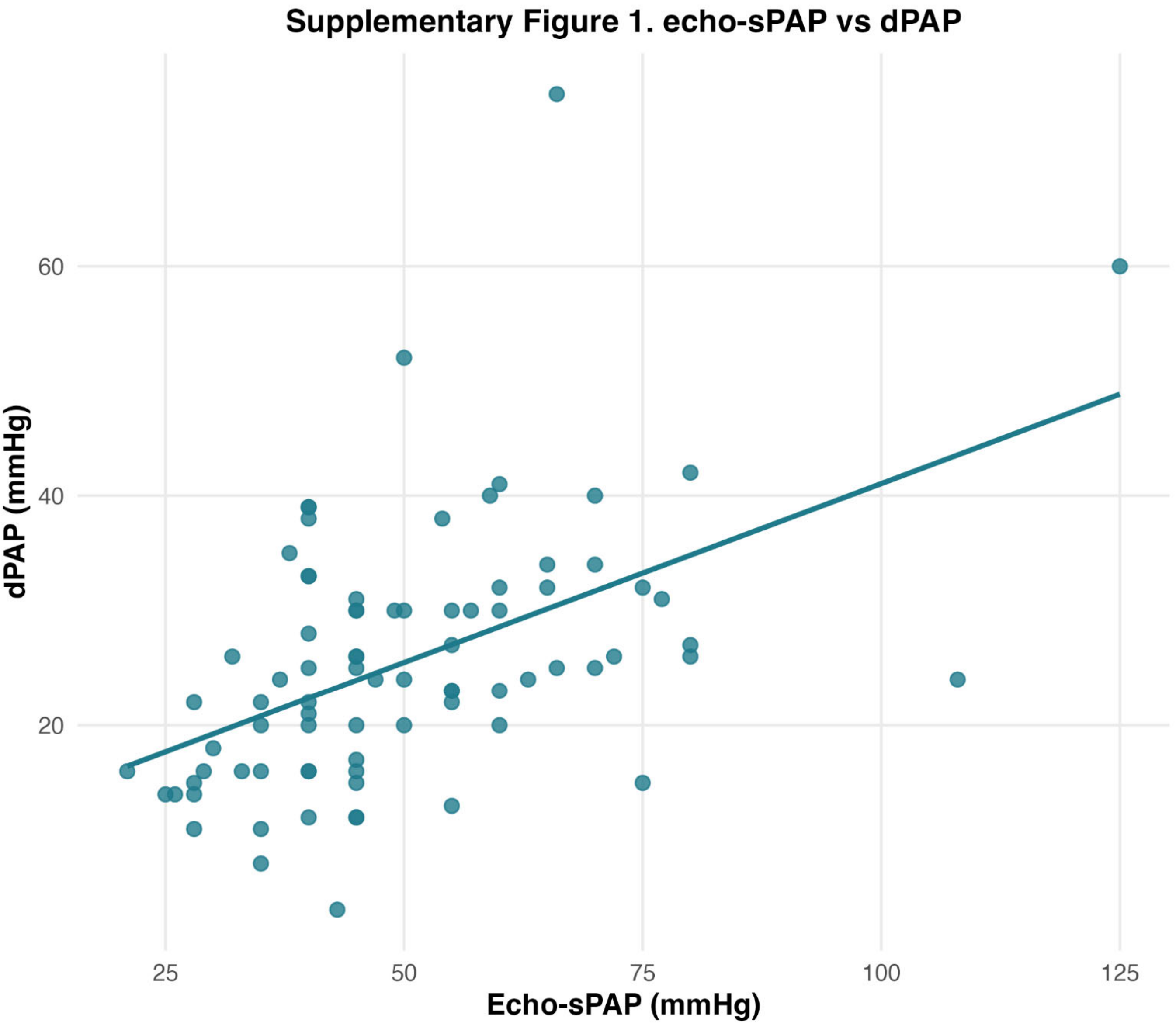

Supplementary Figure S1. Scatter plot of echocardiographic systolic pulmonary artery pressure versus invasive pulmonary artery diastolic pressure. Scatter plot showing the relationship between echocardiographic systolic pulmonary artery pressure (echo-sPAP) and invasive dPAP in the available complete-case subset. The fitted regression line is shown to illustrate the overall direction of association supporting the bridge-model concept.

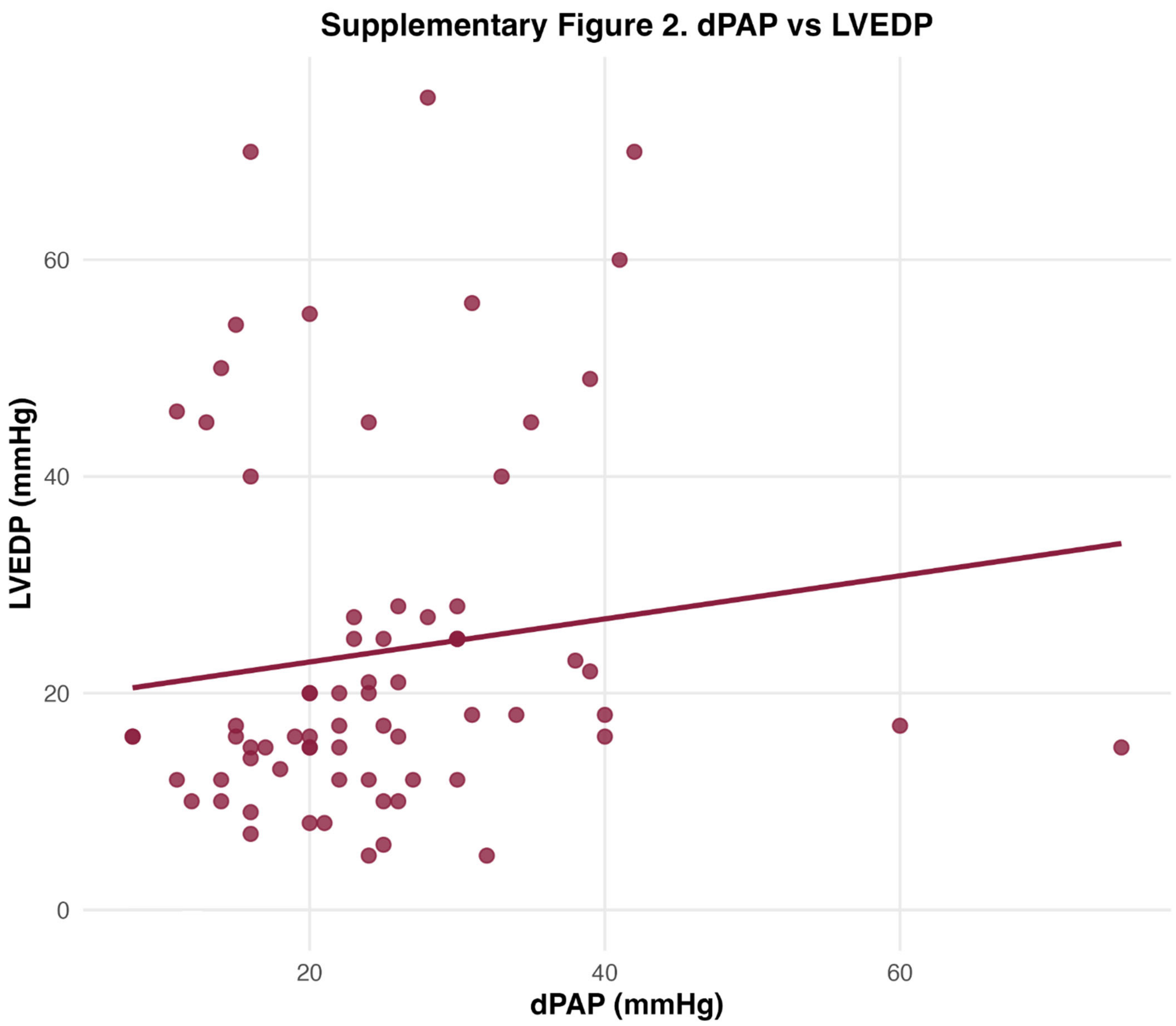

Supplementary Figure S2. Scatter plot of invasive pulmonary artery diastolic pressure versus directly measured LVEDP. Scatter plot showing the relationship between invasive pulmonary artery diastolic pressure (dPAP) and directly measured LVEDP in the available complete-case subset. The fitted regression line is shown to illustrate the overall direction of association between the intermediate invasive pulmonary hemodynamic phenotype and the reference left-sided filling pressure target.

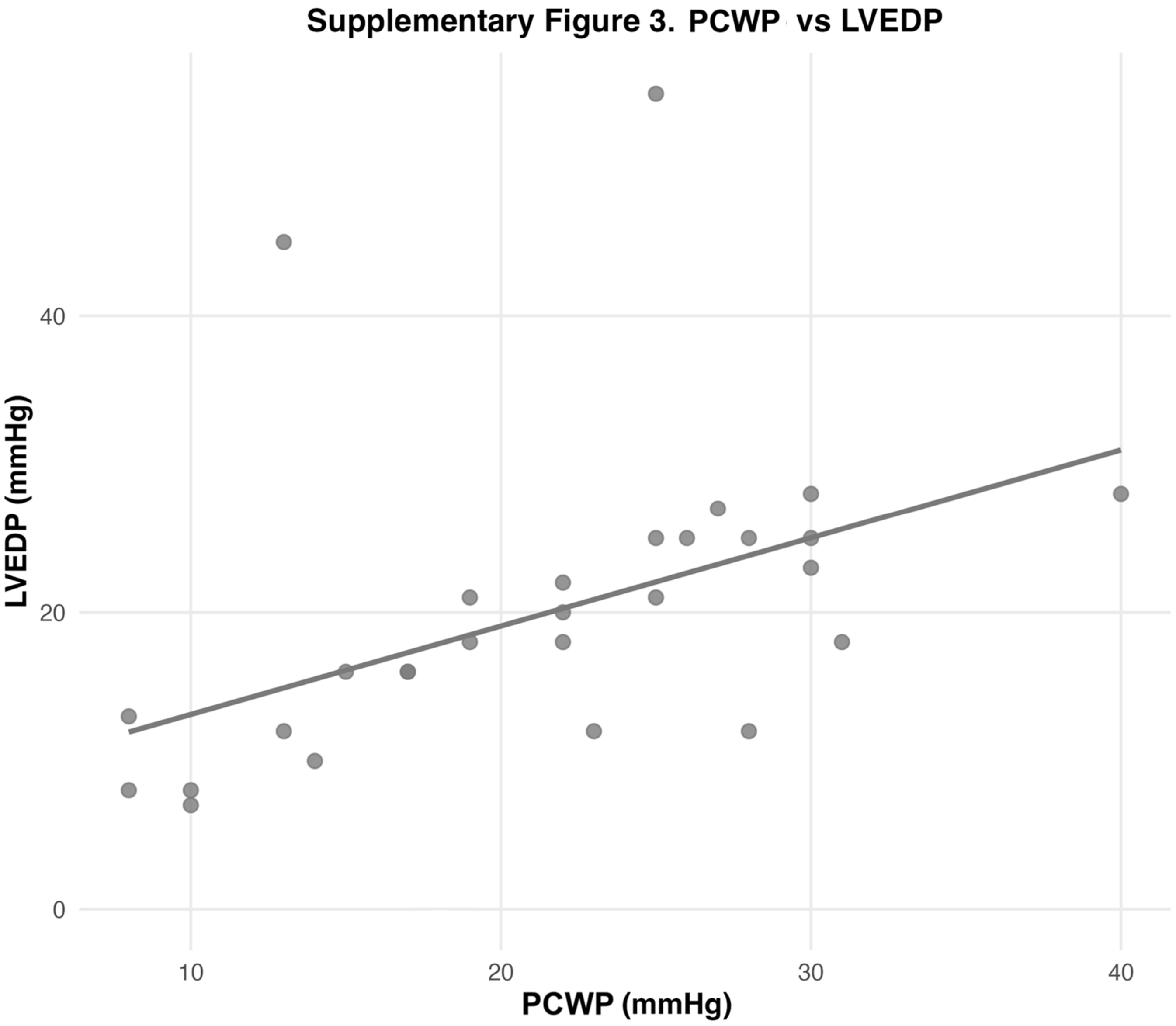

Supplementary Figure S3. Scatter plot of PCWP versus directly measured LVEDP. Scatter plot showing the relationship between PCWP and directly measured LVEDP in the PCWP-available subgroup. This analysis is supportive and limited by the smaller subgroup size.

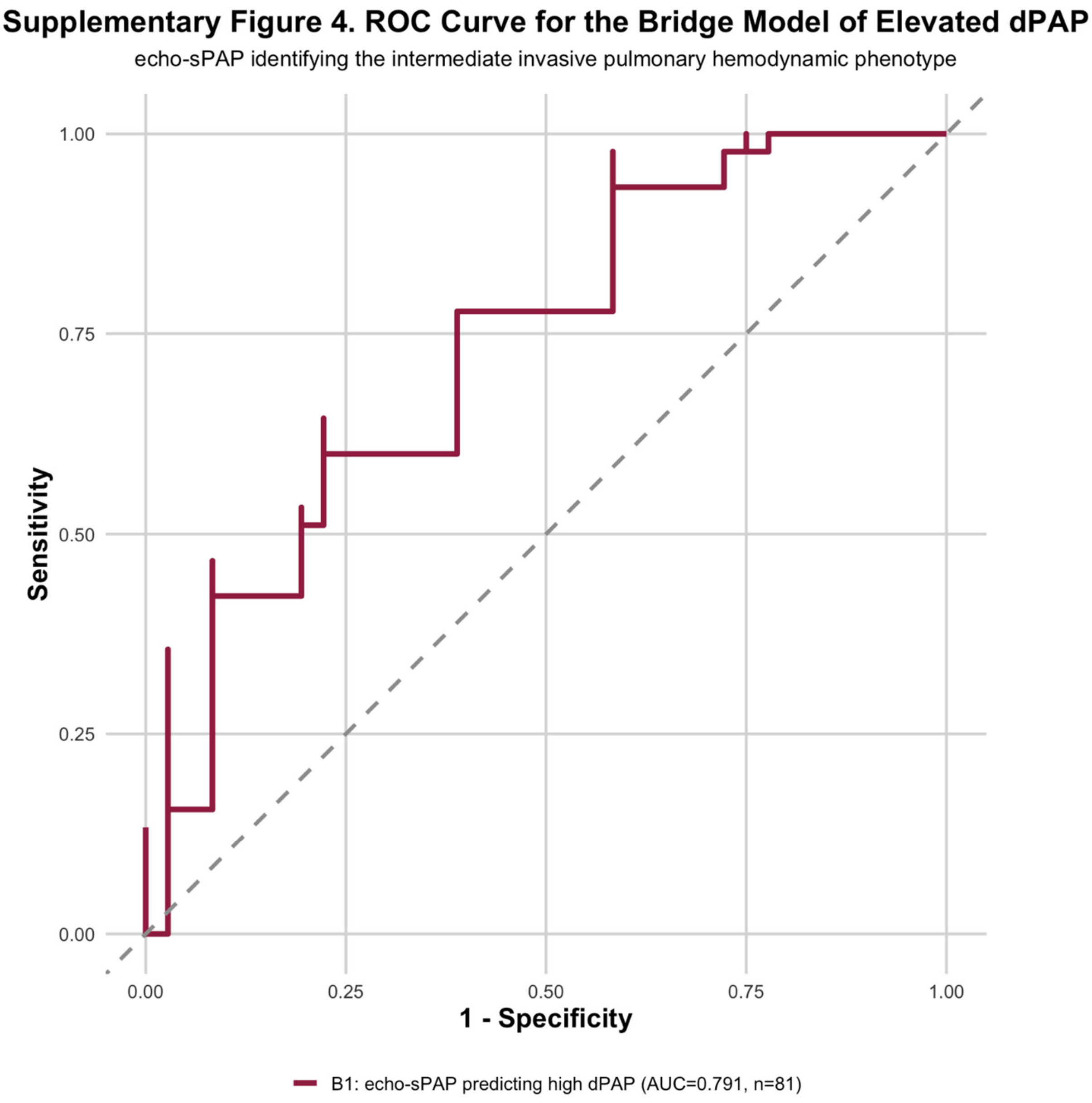

Supplementary Figure S4. Receiver operating characteristic curve for the bridge model of elevated dPAP. Receiver operating characteristic curve for the bridge model using echocardiographic systolic pulmonary artery pressure (echo-sPAP) to identify elevated invasive pulmonary artery diastolic pressure (dPAP ≥24 mmHg). The model showed good discrimination for the intermediate invasive pulmonary hemodynamic phenotype.
